# Comparison of ocular aberrations in three types of rigid gas permeable lenses in keratoconus patients

**Published:** 2020

**Authors:** Shabnam Torkman, Mehdi Khabazkhoob, Haleh Kangari, Abbasali Yekta, Ebrahim Jafarzadehpur

**Affiliations:** * Noor Research Center for Ophthalmic Epidemiology, Noor Eye Hospital, Tehran, Iran; ** Department of Medical Surgical Nursing, School of Nursing and Midwifery, Shahid Beheshti University of Medical Sciences, Tehran, Iran; *** Department of Optometry, Shahid Beheshti University of Medical Sciences, Tehran, Iran; **** Department of Optometry, School of Paramedical Sciences, Mashhad University of Medical Sciences, Mashhad, Iran; ***** Rehabilitation Research Center, Department of Optometry, School of Rehabilitation Sciences, Iran University of Medical Sciences, Tehran, Iran

**Keywords:** ocular aberration, keratoconus, RPG contact lenses

## Abstract

**Purpose:** To determine the effect of different types of Rigid Gas Permeable (RGP) contact lenses on ocular aberrations in patients with keratoconus.

**Methods:** Eighteen eyes of young patients with mild to moderate keratoconus were selected. General ocular examinations such as refraction, visual acuity, and ocular aberrations were performed. Three types of RGP contact lenses, i.e., Boston, Senso Select and Wohlk, were fitted using the cross over method. Repeated measures analysis of variance and Mauchly’s test of sphericity were used to compare the average of residual high order aberrations and visual acuity after fitting each type of lens.

**Results:** Vertical coma was -0.271 ± 0.37 µm before fitting and decreased to 0.081 ± 0.08 µm with Boston, 0.098 ± 0.08 µm with Senso Select and 0.124 ± 0.08 µm with Wohlk contact lens (P-value < 0.0001). The mean RMS (root mean square) for high order aberrations decreased from 0.526 ± 0.43 µm before fitting to 0.256 ± 0.09 µm with Boston, 0.263 ± 0.12 µm with Senso Select, and 0.304 ± 0.10 µm with Wohlk contact lens (P-value= 0.001). The mean RMS for low order aberrations decreased from 1.480 ± 0.78 µm before fitting to 0.703 ± 0.43 µm with Boston, 0.802 ± 0.39 µm with Senso Select, and 0.760 ± 0.45µm with Wohlk (P-value < 0.0001).

**Conclusion:** Despite achieving optimal fit and good visual acuity with these different RGP lenses, in keratoconus patients, their performance is different in reducing ocular aberrations.

## Introduction

Keratoconus is a kind of inflammatory and progressive corneal dystrophy that is usually bilateral and asymmetric. This condition usually leads to high amount of myopic astigmatism [**1**]. The estimated prevalence of keratoconus in the Caucasian population is about 1/ 2000 [**2**]. In a study conducted on the Iranian population selected from Tehran citizens, the prevalence of keratoconus has been estimated at 0.8% [**3**]. Several studies have reported that in eyes with keratoconus, irregular astigmatism, corneal and ocular aberrations individually are larger than in normal eyes [**1**,**4**,**5**]. Due to the localization of conical protrusion of thinned cornea, out of the high order aberrations, third order vertical coma is in negative sign and it is significantly more than in normal eyes [**1**]. Since the quality of retinal images depends on the ocular aberrations, reduction of visual performance in affected patients is clearly described by large magnitudes of high order aberrations. Therefore, reducing ocular aberrations can improve the visual quality in these patients [**6**]. 

There are different methods of keratoconus management according to the intensity of disease progression. Generally, spectacle correction is recommended in early stages, contact lenses are used in mild to moderate stages, and surgery is indicated in severe stages [**7**]. Contact lens fitting remains the basic option for the correction of high amounts of induced irregular astigmatism by keratoconus and could be an appropriate alternative to surgical intervention in moderate cases [**8**,**9**]. 

The most successful lenses are still the corneal rigid gas permeable (RGP) contact lenses [**10**,**11**]. RGP contact lenses are currently the most appropriate option for the correction of high order aberrations like spherical aberration, coma, curvature of field, distortion, trefoil, tetra foil and secondary astigmatism induced by keratoconus [**7**,**12**]. 

Different types of RGP contact lenses, manufacturing and fitting methodologies are the most important factors to choose the suitable contact lenses. Corneal specifications in central and paracentral zones, contact lens design, material, and interactions between the contact lens and the anterior corneal surface are some of the important parameters that influence an optimal fitting. Because of poor centration of the contact lens, corneal scar and staining, and reduced visual acuity are some complications of suboptimal fitting of corneal warpage [**13**,**14**]. 

It has been suggested that contact lens design is the first and the most effective factor in achieving optimal fitting [**14**,**15**]. Currently, different types of RGP contact lens design, containing multicurve and aspheric with unique and variable asphericity in surfaces impose some difficulties regarding the achievement of appropriate fit between practitioners [**7**,**12**]. The purpose of this study was to determine the impact of three types of RGP contact lenses commonly used by Iranian practitioners, i.e., the Boston, Senso Select, and Wohlk, on visual acuity, low and high order aberration, vertical coma, and secondary corneal astigmatism.

## Methods

In this cross-over before-after study, 18 eyes of 11 patients aged 18 to 35 years, with mild to moderate keratoconus, were investigated. The patients were examined by a corneal specialist in the Eye Hospital and were referred to the contact lens clinic with a diagnosis of keratoconus after clinical and paraclinical examinations. The diagnoses of keratoconus were confirmed through imaging modalities. Keratoconus diagnosing was performed according to keratoconus severity score described by CLEK study [**10**]. The reason for choosing mild to moderate keratoconus is that in advanced keratoconus with corneal scars and deformation, corneal contact lenses do not provide acceptable visual acuity and may be intolerable for some patients; therefore, in these cases, more specific designs of contact lenses, like scleral lenses, are recommended [**16**]. The inclusion criteria were a best corrected visual acuity of 20/ 40 or better, no history of ocular diseases including cataract, corneal scarring, corneal grafts, corneal collagen cross linking, and other inflammatory or infectious ophthalmic disorders except for keratoconus. 

Slit lamp biomicroscopy (HAAG-STREIT AG, Switzerland) was performed to assess the extent of keratoconus and presence of central corneal thinning, Vogt’s striae, and Fleischer’s ring. Objective refractive examinations included retinoscopy (HEINE Beta 200, Germany) and auto refractokeratometry (Nidek ark 510a, Aichi, Japan). In addition, keratometry examination was performed using the auto ref-keratometer (Nidek ark 510a, Aichi, Japan). Subjective examination was done with a trial lens set and frame and visual acuity was measured using a Snellen chart (Nidek- 34605-6004-LCD chart, Japan) at 4 m. Ocular aberrations were measured by an auto ref-keratometer with wavefront technology based on the Hartmann-shack technique (Huvitz HRK 8000a, Gyeonggi-do, Korea).

The RGP contact lenses that were studied included the Wohlk (Wӧhlk Contctlinsen, Schӧnkirchen, Germany), Boston (Iran Lens Gostar, Tehran, Iran), and Senso Select (Procornea, Eerbeek, Netherlands). Data were recorded before the first fit. Three types of contact lenses were fitted randomly. The fitting method was based on k-readings parameters and instructions provided by their manufacturers.

Optimal fitting characteristics were verified by the three-point touch fitting method including mild touch on the apex of the cone and two other touch areas on the corneal mid periphery [**7**]. Maximum centration, suitable movement and adequate tear film interchange were evaluated by the fluorescein pattern on slit lamp examinations with cobalt blue light.The maximum time to stabilization for each RGP contact lens was approximately 10-15 min. Then, subjective over-refraction and aberrometry measurements were performed [**17**]. For the corneal curvature to resume its normal shape, 5-10 min was spared after each RGP lens removal [**6**].

All data were analyzed using the Statistical Package for Social Science (SPSS) version 21 (Chicago, IL, USA). The results were compared using repeated measures analysis. Mauchly’s Test of Sphericity was used to analyze the aberration changes among three types of RGP contact lenses. Differences were considered statistically significant when the p-value was smaller than 0.05.

## Results

Eighteen eyes of eleven patients with a mean age of 27.72 ± 5.63 years (range: 18 to 35 years) were included in this study. **Table 1** summarizes the refractive and keratometric profile of the patients, and contact lens characteristics are shown in **Table 2**.

A summary of visual acuity and low and high order aberrations is presented in **Table 3**.

According to **Table 3**, there were no significant differences in visual acuity between RGP lenses, although the Boston contact lens provided the highest visual acuity and the Senso Select and Wohlk contact lenses produced similar values. 

**Table 1:** Clinical profile of all subjects

Measurements	Mean ± SD
Age	27.72 ± 5.63
Refraction (spherical)	-3.12 ± 2.56
Refraction (cylindrical)	-2.83 ± 1.78
Flattest K (mm)	7.46 ± 0.25
Steepest K (mm)	7.10 ± 0.34

**Table 2:** Contact lenses characteristics

RGP contact lenses	Power (D)	Diameter (mm)	Average amount of BC (mm) ± SD
Boston	-3.00	9.60	7.66 ± 0.25
Senso Select	-3.00	9.90	7.78 ± 0.26
Wohlk	-3.00	9.60	7.57 ± 0.14

**Table 3:** Comparison of different parameters before and after fitting three types of RGP lenses

Variables	Mean ± SD Base line	Mean ± SD (Boston)	Mean ± SD (Sens select)	Mean ± SD (Wohlk)	P-VALUE*
Visual acuity (MAR)	1.55 ± 0.29	1.10 ± 0.14	1.08 ± 0.10	1.08 ± 0.10	0.126
Total RMS HOA (µm)	0.526 ± 0.43	0.256 ± 0.09	0.263 ± 0.12	0.304 ± 0.10	0.001
Total RMS LOA (µm)	1.480 ± 0.78	0.703 ± 0.43	0.802 ± 0.39	0.760 ± 0.45	<0.0001
Vertical coma (µm)	-0.271 ± 0.37	0.081 ± 0.08	0.098 ± 0.08	0.124 ± 0.08	<0.0001
SCA (D)	-3.28 ± 2.53	-1.10 ± 0.59	-1.00 ± 0.53	-1.29 ± 0.67	<0.0001
HOA = high order aberrations, LOA = low order aberrations, RMS = root mean square, SCA = secondary corneal astigmatism. *Repeated measures analysis					

The results also showed no significant differences in all aberrations between three types of RGP contact lenses. In terms of high order RMS and vertical coma after fitting RGP contact lenses, the Boston and Wohlk contact lenses displayed the smallest and largest value, respectively. The Boston contact lens also produced the smallest value of low order RMS, whereas the Senso Select contact lens showed the highest value.

The use of the Senso Select and Wohlk contact lenses was associated with minimum and maximum secondary corneal astigmatism, respectively. 

## Discussion

In this investigation, RGP lenses were used in a group of young patients with mild to moderate keratoconus because previous studies reported that the disease progresses in young adults. Therefore, proper prescription is essential in these patients to achieve an appropriate visual acuity and to improve their quality of life. Likewise, in early stages of keratoconus, the manifest refraction of these patients shows an increase in both myopia and irregular astigmatism, commonly ranging between 2.00 and 8.00 D, with absence of parallelism of keratometry mires with a mean corneal power under 52.00 D (**Table 1**) [**7**]. Although RGP lenses are still the best choice to improve visual performance in patients with moderate keratoconus, different available designs of RGP contact lenses and their proficiency of centration in proportion with corneal topography is challenging [**18**]. Our goal in this study was to show that each RGP contact lens reduced aberrations by different mechanisms. 

In this study, the three RGP contact lenses changed coma aberrations, especially vertical coma, in such a way that the direction of vertical coma changed from negative to positive. These directional changes could have influenced the reduction in total HOA RMS, especially in patients with negative vertical coma. These findings are similar to those were reported previously by Choi et al. [**19**]. Better centration with RGP contact lenses could have led to a better reduction of ocular aberrations. In terms of sphericity and asphericity, back optic zone diameter (BOZD) and design of posterior surface of RGP lenses have been mentioned in providing good centration [**20**].

The differences found between three RGP contact lenses can be due to some factors such as corneal centration, the ratio of the base curve radius in relation to the diameter, and tear film volume. Therefore, a suitable lens design also provides optimum fit, reduces residual aberrations, and improves visual acuity [**14**,**21**]. 

As presented in **Table 3**, the subsequent visual loss of keratoconus was corrected with different mechanisms by RGP contact lenses: The Senso Select contact lens showed the best performance in reducing secondary corneal astigmatism in comparison with two other lenses, whereas, it was not successful in reducing low order aberrations. The Wohlk contact lens reduced all parameters to a moderate amount, and the Boston contact lens reduced low and high order aberrations and vertical coma maximally, but it did not reduce secondary corneal astigmatism compared to the Senso Select. Unlike whatever mentioned about aberration changes, the differences in aberrations reduction were not significant between these three types of RGP contact lenses. 

The result of this study showed that the three RGP lenses, i.e., the Boston, Senso Select, and Wohlk, improved visual acuity after optimum fit. The differences in visual acuity improvement were not significant. The mean base curves were different for optimal fit in these RGP lenses that reflect the effect of the design of these contact lenses (**Table 2**).

One of the limitations of this study was that in spite of extensive correspondence and search, the details of these contact lenses were not available, because the details of the lens design are privileged information. In this regard, this study focused on clinical outcomes of each RGP contact lens. A strength of this study was that it compared different clinical parameters pre and post fitting with three RGP contact lenses. Furthermore, each keratoconus patient was fitted with three RGP lenses and compared to him/ herself. 

In conclusion, this investigation showed that after an optimal fit with the Boston, Senso Select, and Wohlk RGP lenses, they all could equally improve visual acuity in keratoconus patients, while their impacts on ocular aberration were different depending on the initial aberrations of their cornea and RGP contact lens design. According to **Table 3**, the performance of these three types of RGP contact lenses in improving the visual acuity was the same and they had the same function in terms of correcting aberrations. In summary, our results indicated that although the design of these three types of RGP contact lenses are different, all of them improved visual performance by reducing ocular aberrations and there is no preference in selecting the best lens for keratoconus patients.

**Conflict of Interest**

None of the authors has any ﬁnancial interest in the rigid gas permeable contact lenses mentioned.

**Financial Support**

None.
